# Layer-by-layer assembly of nanotheranostic particles for simultaneous delivery of docetaxel and doxorubicin to target osteosarcoma

**DOI:** 10.1063/5.0180831

**Published:** 2024-02-29

**Authors:** Liam Desmond, Simone Margini, Emilio Barchiesi, Giuseppe Pontrelli, Anh N. Phan, Piergiorgio Gentile

**Affiliations:** 1 School of Engineering, Newcastle University, Newcastle upon Tyne, United Kingdom; 2 Department of Architecture, Design and Urban Planning, University of Sassari, Alghero, Italy; 3 Istituto per le Applicazioni del Calcolo-CNR, Rome, Italy

## Abstract

Osteosarcoma (OS) is a rare form of primary bone cancer, impacting approximately 3.4 × 10^6^ individuals worldwide each year, primarily afflicting children. Given the limitations of existing cancer therapies, the emergence of nanotheranostic platforms has generated considerable research interest in recent decades. These platforms seamlessly integrate therapeutic potential of drug compounds with the diagnostic capabilities of imaging probes within a single construct. This innovation has opened avenues for enhanced drug delivery to targeted sites while concurrently enabling real-time monitoring of the vehicle's trajectory. In this study, we developed a nanotheranostic system employing the layer-by-layer (LbL) technique on a core containing doxorubicin (DOXO) and in-house synthesized carbon quantum dots. By utilizing chitosan and chondroitin sulfate as polyelectrolytes, we constructed a multilayered coating to encapsulate DOXO and docetaxel, achieving a coordinated co-delivery of both drugs. The LbL-functionalized nanoparticles exhibited an approximate size of 150 nm, manifesting a predominantly uniform and spherical morphology, with an encapsulation efficiency of 48% for both drugs. The presence of seven layers in these systems facilitated controlled drug release over time, as evidenced by *in vitro* release tests. Finally, the impact of the LbL-functionalized nanoparticles was evaluated on U2OS and Saos-2 osteosarcoma cells. The synergistic effect of the two drugs was found to be crucial in inducing cell death, particularly in Saos-2 cells treated with nanoparticles at concentrations higher than 10 *μ*g/ml. Transmission electron microscopy analysis confirmed the internalization of the nanoparticles into both cell types through endocytic mechanisms, revealing an underlying mechanism of necrosis-induced cell death.

## I. INTRODUCTION

Theranostics is a promising emerging field of medicine by combining “therapeutics” and “diagnostics,” where drugs diagnostics, treatment, and monitoring are combined. This has proven to be time and money effective, and the ability to bypass some of the undesirable biological effects that may arise when these strategies are employed separately.^1^ Nanotheranostic medicine, using nanoparticles (sizes 10–1000 nm), can operate more efficiently than standard theranostics medicine ranging from dendrimers, nanocrystals, and liposomes, such as capabilities in an all-in-one single platform, which include sustained/controlled release, targeted delivery, higher transport efficiency by endocytosis, stimulus responsive agent release, synergetic performance, multimodality diagnosis and/or therapies, and quality performances.^2^

Particularly, nanotheranostics consist of macromolecular materials/polymers in which the diagnostic and therapeutic agents are adsorbed, conjugated, entrapped, and encapsulated for diagnosis and treatment simultaneously at cellular and molecular level.^3^ Currently, various nanodrug systems have been developed and employed to improve the efficacy, safety, physico-chemical properties, and pharmacokinetic/pharmacodynamic profile of pharmaceutical substances.^4^ Despite this clear advantage, the drawbacks, such as the toxic potential of nanodrugs, since they often exhibit *in vitro* and *in vivo* cytotoxicity, oxidative stress, inflammation, and genotoxicity, have signified that a new and less dangerous method of nanodrug delivery must be established.^5^ There is continuous research into the synthesis and applications of colloidal micro- and nano-spheres in the use of drug delivery systems, since their intrinsic properties, e.g., diameter, can be tuned and they have many applications in industry including drug delivery.^6^ Until now, only amorphous silica and some colloidal spheres can be routinely prepared with satisfactory narrow size distributions, characterized by inert surfaces, which make surface modification almost unavoidable before use.^7^ Then, coating the spheres with noble-metal nanoparticles, oxide nanoparticles of semiconductor-quantum dots could endow the spheres with specific catalytic, magnetic, electrical, optical, or optoelectronic properties and widen the utility of them. However, the use of metal-based nanoparticles has resulted in aggregation and cellular toxicity of these particles which limit their clinical application in cancer therapy.^8^ Various nanoparticles, including iron oxide nanoparticles, carbon nanotubes, and quantum dots, have been extensively studied to determine whether they could be forwarded as a cancer therapeutic approach.^8^ However, the development of green, sustainable, nontoxic, and high-performance nanoparticles for cancer related diagnosis and treatment is an area of considerable interest owing to the drawbacks of current diagnostics/treatments, such as (1) tumor cells can be very resistant to conventional therapies; (2) the target area is susceptible to damage from conventional therapy and has a very limited capacity to repair itself; and (3) many drugs cannot cross the blood barrier to act on these tumors and/or have unacceptable systemic toxicities.^4^

Furthermore, the ability of bioimaging into nanocarriers is crucial to provide real-time and direct observation for specific molecular events and biological pathways. This can help design a strategy for the enabling of effective cancer treatment management both *in vivo* and *in vitro.*^9^ In this work, we have investigated the potential of in-house synthesized carbon quantum dots (CQDs) from biomass as imaging nanoprobes. A recent study of the use of CQDs in bioimaging was carried out by Huang *et al.*^10^ to emphasize the significance of CQDs in *in vivo* optical bioimaging studies. Specifically, a nude mouse was inoculated with Smmc-7721 tumor cells, and the optical imaging of the CQDs (generated from wheat straw, a biomass waste) was investigated by intravenous injection of 200 *μ*l of the synthesized CQDs via the tail vein. Three hours after the injection, the CQDs circulated in the mouse's body and within 12 h, it was observed that the CQDs stabilized almost exclusively at the location of the tumor, with no fluorescence signals detected in the organs of the heart, lung, and spleen.

For the manufacturing of nanotheranostic systems, one of the most promising approach consists in the layer-by-layer (LbL) self-assembly, which represents an alternative surface modification technique to Langmuir–Blodgett deposition and self-assembly monolayers methods. LbL works on the alternating exposure of a charged substrate to solutions of positively and negatively charged polyelectrolytes and is an effective as well as economic process to fabricate well-organized multilayers at nanometer scale.^11^ Furthermore, LbL assembly technology allows a precise control of the coating properties attainable, i.e., thickness control, and is an environmentally friendly and low-cost process low-cost manufacturing and versatile for coating all available surfaces allowing the incorporation and controlled release of any types of biomolecules/drugs.^12,13^ The application of the LbL to create nanosystems for the incorporation of a QD signal amplification tag was reported for the first time by Yu and Pishko,^14^ by alternating attachment of streptavidin and biotin-conjugated cadmium sulfide (CdS) QDs onto the surfaces of nanosized polystyrene particles. Furthermore, LbL self-assembly approaches have been successfully applied in graphene nanosheets (GNs)-CdS QDs composite films (where positively charged GNs-PAH and negatively charged CdS QDs were employed as nano-building blocks) and CdSe/zinc sulfide (ZnS) QD assemblies (where a dithiol linker was used to make multilayers of CdSe/ZnS QDs, while in the second biotin- and streptavidin-conjugated CdSe/ZnS QDs were used to make multilayer constructs).^15^ Moreover, LbL process has been successfully used in creating uniform coating of eco-friendly red-emissive hollow nitrogen-doped with a quantum yield comparable to Cd/Pb QDs and tunable characteristics depending on the characteristics required for certain applications.^16^ This recent example clearly evidence that CQDs can successfully undergo LbL deposition techniques to generate an overall nanosystems akin to standard QDs, as more advantageous due to their lack of toxicity.^10^

This work aimed to investigate the application of the LbL assembly to develop nanometer-sized theranostics systems loading a payload formed by two chemotherapeutic drugs [doxorubicin (DOXO) and docetaxel (DTX)] and in-house synthesized CQDs (as imaging nanoprobes) for treating osteosarcoma (OS). The processing parameters for the development of these nanotheranostic systems have been discussed with particular attention on the *in vitro* drug release tests, studying the drugs individual or co-delivery supported by a developed computational model, to achieve a controlled a timely payload release capable to increase the efficacy of the intended treatment against two osteosarcoma cell lines, Saos-2 and U2OS.

## II. RESULTS AND DISCUSSION

All nanoparticles formed by the LbL assembly process were generated by employing chitosan (CH) and chondroitin sulfate (CS) as polyelectrolytes. Following the LbL assembly process, three types of nanoparticles were manufactured consisting of the hydrophilic CQDs as core functionalized with the seven layers: (1) without drugs (coded as CQD_7L), (2) with DOXO (coded as CQD_7LD), and (3) with DOXO and DTX drugs (coded as CQD_7LDD). Also, CQDs embedded with 1 CH layer containing DOXO (coded as CQD_1LD) were examined as control to verify the success of encapsulation of the CQDs as cores of the nanoparticles. The ζ-potential charge of the prepared solutions was found experimentally to be −13.7 ± 0.9 mV for the CQDs, +40.1 ± 0.5 mV for CH, and −20.1 ± 2.3 mV for CS, respectively, which were determined at pH 5. This was consistent with previous LbL assembly reported in the literature where CH and CS were used at pH 5 for the manufacturing of a multilayered coating.^14–16^

### A. Encapsulation of the CQDs as core of the nanoparticles (CQDs_1LD).

**FIG. 1.**
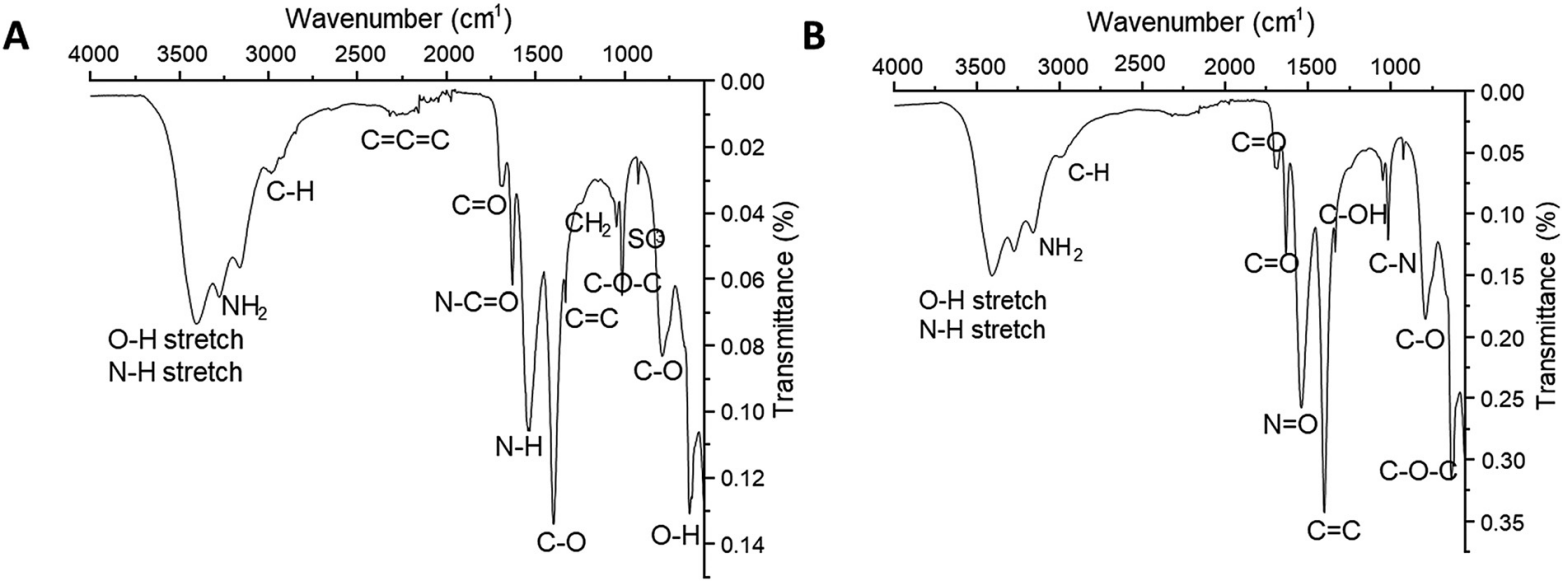
FTIR-ATR spectra of (A) CQDs_1L and (B) CQDs_7LDD nanoparticles.

For the LbL assembly process, hydrophilic and negatively charged CQDs were employed as core for the formation of fluorescent nanoparticles. To confirm the successful encapsulation, Fourier transformed infrared spectroscopy (FTIR)-attenuated total reflectance (ATR) analysis was performed. Figure 1(a) shows the spectrum of the successful formation of the cores. Particularly, the analysis confirmed peaks belonging to the initial CQDs, which were O–H ∼ 3405 cm^−1^, NH_2_ ∼ 3273 cm^−1^, C–H ∼ 2988 cm^−1^, C–O ∼ 1100 cm^−1^, C=O ∼ 1600 cm^−1^, and C=C ∼1300 cm^−1^, consistent with established infrared peaks attributed with previously synthesized CQDs.^17^ The latter two peaks correspond to the negative moieties of the CQDs that account for the π–π^*^ electronic transitions that give rise to the fluorescence of the CQDs.^18^ Despite the fact that the successful addition of the CH layer would result in encapsulation of the CHBOCQDs core, it has been established that the average polyelectrolyte layer deposited on this type of nanosystems are approximately 2–3 nm in thickness, measured for each deposition step,^19^ and FTIR-ATR has a surface penetration of 0.664 *μ*m.^20^ Therefore, it is reliable with FTIR-ATR to study the bonds of the core, and this analysis is capable of measuring all the nanolayers in a multilayer system.^21^ Moreover, the peaks that correspond to the positive CH polyelectrolyte were evident in the sample: NH_2_ ∼ 3435 cm^−1^, CH_2_ ∼ 1310 cm^−1^, and C–O–C ∼ 1010 cm^−1^, which are consistent with previous FTIR-ATR results reported in the literature.^22^ The peaks that correspond to the presence of DOXO in the sample were O–H ∼ 3405 cm^−1^, N–H ∼ 3300 cm^−1^, C–H ∼ 2988 cm^−1^, C–O ∼ 1100 cm^−1^, C=O ∼ 1600 cm^−1^, C=C ∼ 1300 cm^−1^, and C–O–C ring bend at 700 cm^−1^ consistent with previous infrared analysis of DOXO.^30^ It is important to note that several of the bonds were present in two or all three of these components. While this established the presence of CQDs, CH, and DOXO in the sample, some other considerations can be done on the mutual interaction between DOXO/CH and CQDs/CHT. Indeed, it has been established that deposition of CH onto CQDs arises by ion-dipole forces that occur between the two components, namely, NH_2_ and SO_3_ dipole interactions.^23^ These NH_2_ and SO_3_ bonds were present on the spectra (∼3270 and ∼1020 cm^−1^, respectively) [Fig. 1(a)], which validated that CQDs and CH have successfully bounded together, confirming the successful deposition of the polycation. Furthermore, the confirmation that the DOXO has successfully incorporated into the CH polyelectrolyte layer in solution was evident from a N–C=O bond,^24^ being clearly visible at ∼1580 cm^−1^ in the infrared spectra.

### B. Physico-chemical characterization of the LbL-functionalized nanoparticles

Following the successful simultaneous deposition of the first polyelectrolyte layer onto the CQD core and encapsulation of DOXO into the polyelectrolyte layer, the LbL-assembly process was carried out to coat the remaining layers. FTIR-ATR was analyzed following the subsequent addition of the six remaining layers to create CQDs_7LDD nanoparticles, which was incorporating both drugs [Fig. 1(b)]. The interaction between the CH and CS polyelectrolytes was evident due to ion dipole forces, which are H bonds and coulombic forces.^25^ The H bonds arose between OH of CH and COOH of CS at 795 cm^−1^ as a C–O bond. Furthermore, the coulombic force arises between the NH_3_ of CH and OSO_3_ of CS, present as a N=O and C–N bond at 1547 and 1020 cm^−1^, respectively. This was also observed in the samples CQD_7L with no drugs incorporation (Fig. S3). Furthermore, the C–O–C bond present at 695 cm^−1^ denoted the interaction between DTX and CS,^26^ while the interaction between CH and DOXO is present by a N–C=O bond at 1580 cm^−1^ (Refs. 27 and 28) in Figs. 1(b) and S3. The investigation into this FTIR-ATR spectra confirmed that the LbL process has successfully garnered an assembly between the opposing layers and the drugs incorporated.

**FIG. 2.**
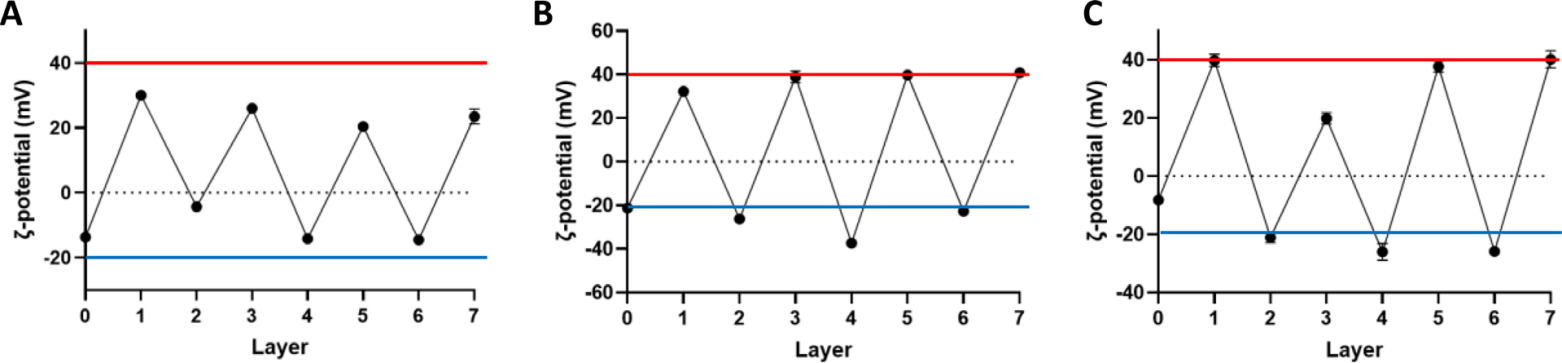
ζ-potential measurement as a function of the layer number for (A) CQD_7L, (B) CQD_7LD, and (C) CQD_7LDD. For each measurement, the values are represented as average ± standard deviation (n = 3). The red line represents the saturation value of the CH layers, while the blue line represents the saturation value of the CS layers.

The validation of successive polyelectrolyte layer addition to develop a system with seven layers was also established by ζ-potential measurements taken upon the addition of each layer (Fig. 2). For the samples CQD_7L without incorporation of the drugs, the ζ-potential measurements changed from an initial value −13.7 ± 0.9 mV (core of CQDs) to +30.0 ± 1.3 mV after deposition of the first layer. Then, for the subsequent six remaining layers deposited onto the nanoparticle precursor, the ζ-potential values oscillated from −4.3 ± 1.0 mV (layer 2), +26.0 ± 1.1 mV (layer 3), −14.2 ± 1.4 mV (layer 4), +20.4 ± 1.5 mV (layer 5), −14.5 ± 0.5 mV (layer 6), and +23.5 ± 2.3 mV (layer 7), respectively [Fig. 2(a)]. As established by previous LbL assembly processes,^29^ the LbL assembly process is the consecutive deposition of oppositely charged polyelectrolytes, relying on strong electrostatic interactions between the polycations and polyanions layers. The resulting ζ-potential at each layer showed that there was no presence of the previously added polyelectrolyte, confirmed by uniformity of the peaks in the ζ-potential measurement.^30^ This surface charge inversion after every deposition step is an essential precondition for LbL assembly of polyelectrolytes.^31^ Then, after the incorporation of DOXO into the nanolayers (CQDs_7LD), comparable results in terms of ζ-potential charges were detected, reaching similar values for each polycationic and polyanionic layer saturation (ranging between +40.8 ± 2.2 mV and −37.4 ± 1.4 mV) [Fig. 2(b)]. It was observed that the saturation of the anionic layers was present in all the layers, while the cationic layers had reached this saturation by layer 5. Then, with the addition of DTX dissolved in CS polyelectrolyte solution (CQD_7LDD), the alternation of charge upon addition of each successive polyelectrolyte layer was clearly obtained after incorporation of both DOXO and DTX, ranging from +40.2 ± 2.9 to −25.9 ± 1.7 mV, as outlined in Fig. 2(c). Moreover, when comparing these results with the CQDs_7L nanoparticles, the encapsulation of the drugs into the system facilitated stabilization of the polyelectrolyte deposition, evident by the positive layers having a higher ζ-potential and for the negative layers, a more negative charge was observed. This could be attributed to evidence outlined in previous LbL assembly, which outlined that upon the addition of DOXO/DTX to polyelectrolyte layers, some of these molecules were localized in the porosities created into the coated surface of the nanoparticles, thus ensuring a greater structural stability of the deposited CH/CS layer and the subsequent layers.^32^ Furthermore, as outlined by previous investigation into DOXO employment,^33,34^ the accumulation of DOXO drug molecules in the porosities of the CQDs cluster ensures structural stability, further stabilizing the polyelectrolyte deposition, inducing an increase in surface charge and saturation of all of the subsequent layers. This denoted that the deposition of pre-layers before layer deposition to stabilize the structure is not required.^35^

After freeze-drying all the nanoparticles types, the process yield for CQD_7L was 11.0 ± 0.4%, while for CQD_7LD was 18.3 ± 0.3% and the CQD_7LDD showed a value of 30.3 ± 0.1%. The yield increase in the nanoparticles encapsulating both DOXO and DTX can be due to the encapsulation of the two drugs made the nanoparticles heavier, preventing their dispersion (thus their loss) during the washing and centrifugation steps. However, the quantity of resulting functionalized nanoparticles is significantly low for all types obtained. This is mainly caused by the method used for their manufacturing, which consists in a manual process that is characterized by various collection, washing and centrifuging processes that can lead a lost in the amount of nanoparticles during each step.

**FIG. 3.**
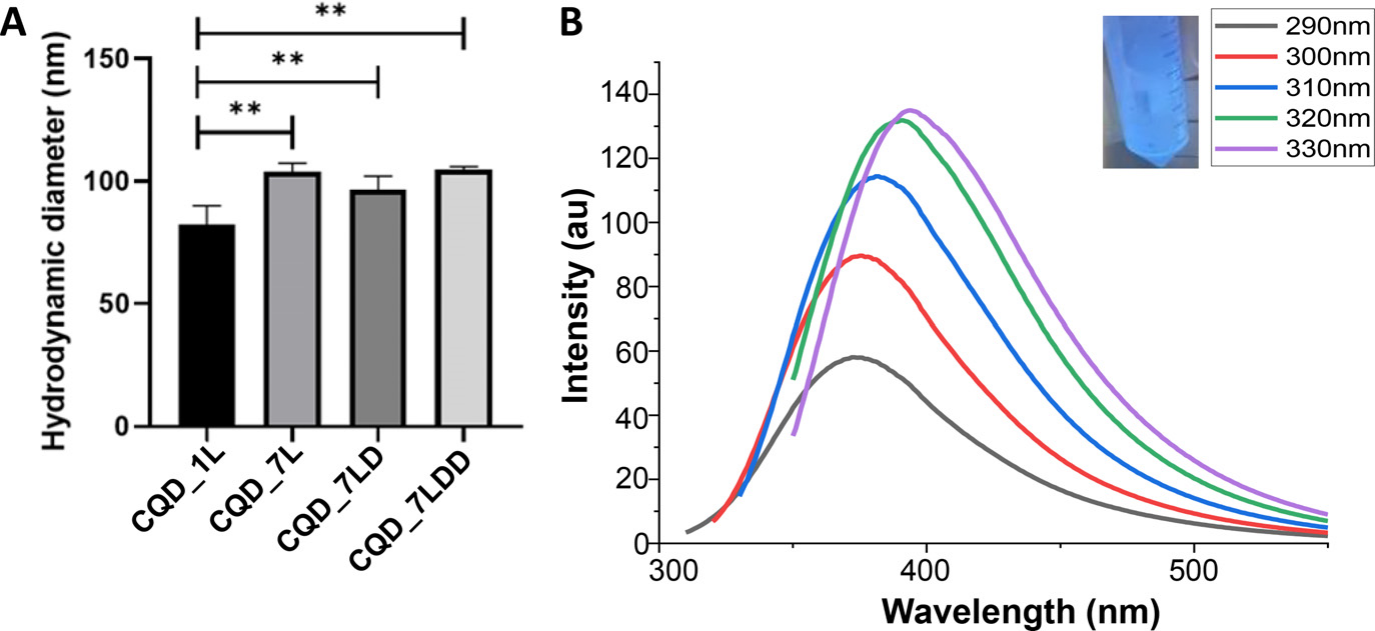
(A) DLS readings for the hydrodynamic diameter/size (nm) of CQD_1L, CQD_7L, CQD_7LD, and CQD_7LDD, with the statistical differences between the different nanoparticles outlined by ^*^. (B) PL spectra of the CQDs_7LDD at a range of excitation wavelengths, inset is the CQDs_7LDD under 365 nm UV light and the resulting color it displays (blue).

Dynamic light scattering measurements were performed to assess the size of the resulting nanoparticles (Fig. 3). CQD_1L exhibited a size of 84.2 ± 7.7 nm, CQD_7L had an average size of 103.9 ± 3.4 nm, CQD_7LD displayed a size of 96.6 ± 5.5 nm, and CQD_7LDD had a size of 104.6 ± 1.3 nm. All the manufactured nanoparticles with seven layers had a size of around 100 nm, which is suitable as nanotheranostic systems. Then, it can be also observed that after the addition of the first layer, the addition of six subsequent layers did not cause the overall size to increase by a substantial amount (approximately 12–30 nm). Previous literature^19,29^ corroborated this behavior and reported that the addition of layers has not added much increase in terms of size, as the size of each individual layer was measured approximately 10 nm or less. Furthermore, the comparison between the drug incorporated and drug free nanoparticles revealed by ANOVA statistical analysis, showed that there was no statistical significance between them, inferring that the encapsulation of drugs did not affect the size of the overall nanoparticles. Furthermore, drug addition to the layers of nanoparticles usually develops a self-assembled system with a size of approximately 100 nm. The trait of this size enables that the system has a high surface area to volume ratio.^36^ Not only that, but the shape derived is usually spherical, which optimal for drug delivery and pharmaceutical as has the highest surface area-volume ratio, helping the further cellular uptake. Previous nanoparticles that have a similar LbL composition reported self-assembly of a similar size (100 nm) with DTX^37^ and DOXO^38^ incorporated into LbL-functionalized nanocarriers for selectively controlled drug delivery.

**FIG. 4.**
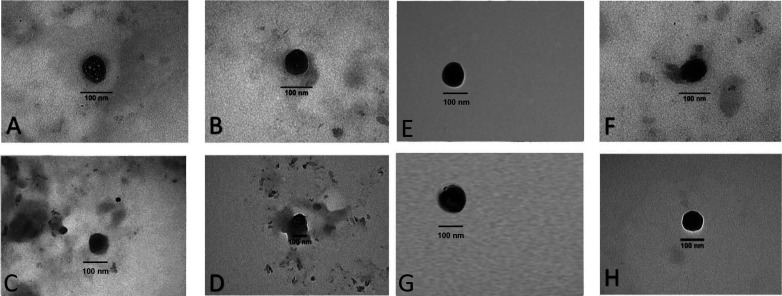
TEM images of CQD_1L (A) and (B), CQD_7L (C) and (D), CQD_7LD (E) and (F), and CQDs_7LDD (G) and (H) at different magnifications.

To better understand the results obtained and verify their correctness, the structures mentioned above were observed by transmission electron microscopy (TEM) analysis, as shown in Fig. 4, where the size of the nanoparticle increased from 84.2 ± 7.7 nm [Figs. 4(a) and 4(b)] to 104.6 ± 1.3 nm [Figs. 4(c) and 4(d)], showing approximately 20 nm size increase, after deposition of seven layers. As established previously,^39^ the individual layers can account for less than 10 nm each of the size each, which was further corroborated by^40^ who summarized from previous nanoparticle by similar LbL assembly processes, that the value of the overall size did not increase by a huge amount upon polyelectrolyte layer deposition, with values as low as 2–3 nm recorded for each deposition step. TEM images of CQD_7LD and CQD_7LDD [Figs. 4(e)–4(h)] displayed a size to 100 nm corroborated by the simultaneous DLS measurements of each system. All the nanoparticles appeared to present a spherical and uniform morphology, while displaying a slight surface roughness, which are all requisites observed in this type of nanoparticle system.^41^

An initial fluorescence test was carried out on the CHBCOQDs_7DD to determine that the overall system was fluorescent, so able to emit light to be detected after specific excitation. This is related to the final aim of the manufactured nanotheranostics to be imaged when implanted *in vivo* to follow their path reaching the tumoral tissue.^48^ This test would also ascertain that the CQDs had successfully remained intact/been successfully encapsulated into the core during the multiple LbL deposition steps, and that the multilayered coating was not affecting their ability to emit.

### C. Fluorescence measurements and bioimaging of the manufactured nanoparticles

All the LbL-functionalized nanoparticles generated were tested for their fluorescence properties. The nanoparticles were dissolved in a water solution and excited with a wavelength of 365 nm. Figure 3(b) (inset) shows a blue color in the solution, confirming the presence of the CQDs within the nanoparticles as reported in similar systems in the literature where QDs incorporated into nanosystems displayed the same fluorescent color as the QDs out of the system as on own, forming bioluminescent nanoparticles.^42–44^ Following this, PL spectra of the CQDs_7DD was recorded and outlined in Fig. 3(b) to determine that this overall nanoparticle was capable to act as bioimaging probe. The excitation dependent photoluminescence emission is displayed, which can be attributed to the quantum confident effect of the different functional groups inducing different emission states.^45^ When excited at 290 nm, the samples showed an excitation peak at 360 nm and exhibited an increase in photoluminescence intensity with a rise in the excitation wavelength. The emission wavelength showed a red shift of 50 nm when the excitation was varied from 290 to 330 nm, which can be attributed to the degree of the quantum confinement effect,^46^ caused by different functional groups inducing different emission states.^45^ This result established that the overall nanoparticles containing both drugs were capable of performing bioimaging for nanotheranostic applications.^47^

### D. Drug release tests.

**FIG. 5.**
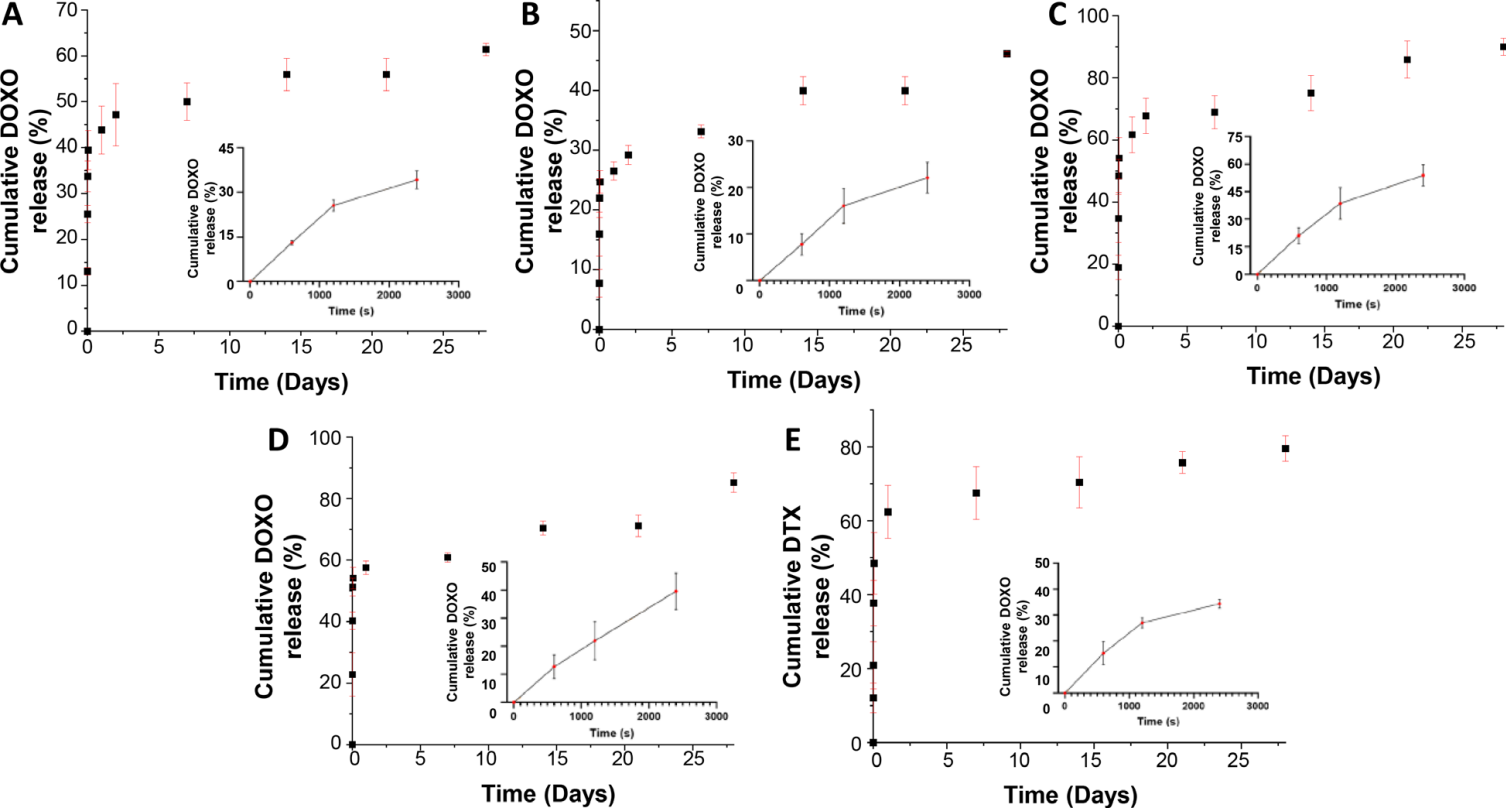
Cumulative drug release tests up to 28 days of immersion in PBS at pH 7.4: DOXO release curve from CQD_1L (A), CQD_7L (B), CQD_7LD (C), CQD_7LDD (D), and DTX release curve from CQD_7LDD (E). The inserts in each figure represent the release of the DOXO during the initial 3000 s.

Figure 5(a) shows the drug release behavior of the nanoparticle CQD_1L was investigated over the 28-day period, to confirm the successful DOXO incorporation and its ability to be released from the core. The analysis revealed that ∼44 ± 5% of the DOXO was released within 24 h. After 7 days, an overall release of 50.0 ± 4.1% was recorded with a cumulative release reached 61 ± 1% after 28 days. The burst release observed initially can be attributed to DOXO diffusion within the layer or a specific phenomenon occurring at pH of 7.4.^48^ This is in accordance with previously synthesized CH-based nanoparticles that exhibited a burst in drug release within the first 24 h due to the trapping capacity of CH and surface adsorption.^49^ Following the initial 24 h, the subsequent drug release profile over the next 7 days appeared to be more uniform with a linear release. Then, from day 7 to day 28, the release values tended to plateau, indicating a steady but minimal release of DOXO (∼18% increase), that can be due to the remaining DOXO molecules becoming trapped within the polyelectrolyte layer without the ability to be released. Encapsulation of drugs within the polyelectrolyte matrix is essential for controlled release from nanoparticles,^50^ but it can also hinder the straightforward release of the drugs.^51^ Moreover, plateauing of the release could also be attributed to the permeabilization of the membrane upon incorporation of the nanoparticles into the target site, allowing the diffusion of low-molecular-mass ions while the macromolecular drug substances remain trapped within the particle.^52^ After day 21, DOXO increased its release rate, reaching a final value of 61 ± 1% at day 28. This behavior can be attributed to the degradation of the layer and the release of the embedded payload into the surrounding environment,^53^ where the degradation could be influenced by the pH shift observed when the loaded nanoparticles (functionalized at pH 5) enter the cellular environment with a pH of approximately 7.4 through endocytosis.^54^ Then, the subsequent nanoparticles were coated with multiple layers. Figure 5(b) illustrates the percentage of DOXO drug release from the nanoparticle CQDs_7L to verify if the drug release mechanism varied depending on the number of deposited layers. After 28 days, the amount of DOXO released was ∼46 ± 1%, indicating that the polyelectrolyte shell acted as a barrier for the release of loaded drugs, resulting in increased release time. Additionally, as shown in Figs. 5(a) and 5(b), the burst release occurred within the first 24 h. However, compared to CQD_1L, the burst release in CQD_7L was reduced up to 27 ± 2%, confirming that the presence of additional layers ensured a more controlled drug release by creating a larger barrier for drug diffusion. Moreover, the degradation of the layers is considered a contributing factor to the high release observed after 28 days as mentioned before. Specifically, from day 14 until day 21, the DOXO release plateaued at 40 ± 2%. However, from day 21, the drug release value increased to 46.2 ± 0.3% by day 28, representing an additional 6.2% increase after plateauing.

The next step in the analysis involved measuring the cumulative release of DOXO from the CQD_7LD with the presence of DOXO in the polycationic layers [Fig. 5(c)]. The seventh layer did not contain any drugs, as previous research^55^ demonstrated that adding drugs to the outer layer would result in their diffusion into the surrounding environment before cellular uptake. This principle was followed in the synthesis of all subsequent nanoparticles. The CQD_7LD nanoparticles exhibited a burst release, increasing from 44 ± 5% to 62 ± 6% compared to CQD_1L, indicating a higher content of encapsulated DOXO in the system.^56^ Particularly, after 2 h, approximately 54 ± 7% of the drug was released, whereas CQD_1L and CQD_7LD released 47 ± 7% and 29 ± 2%, respectively. This can be attributed to the encapsulation efficiency (EE) of the nanoparticles and the drugs, where higher EE results in slower drug release due to stronger binding affinity between the drugs and the nanoparticles.^56^ The analysis revealed that CQD_1L had a DOXO EE of 69 ± 2%, while in CQD_7L, the EE reduced up to 26 ± 2%. After the 28-day measurement period, almost complete drug release (90 ± 3%) was observed from the CQD_7LD system, reaching a plateau before the degradation of the nanoparticle materials. This is similar to previous systems that showed higher than expected drug release, such as carbon-based nanoparticles used as carriers for sodium ibuprofen release.^57^

Finally, the release of DOXO and DTX from the CQD_7LDD has been assessed to verify CHBOCQDs_7DD, whether both drugs could be released simultaneously at a controlled rate [Figs. 5(d) and 5(e)]. The presence of DTX did not significantly influence the DOXO release as also reported in a previous work.^58^ However, between day 22nd and 28th, there was a significant increase in DOXO release, indicating a higher degradation rate in the presence of both drugs compared to the CQD_7LD. This could be attributed to factors, such as pH shock, dehydration, and the nonspecific distribution of DTX, which can cause disorganization and degradation of the nanoparticle.^59^

Regarding the release of DTX, a burst release of approximately 62 ± 7% was observed within the first 24 h, higher than the burst release of DOXO from the same nanoparticle. While the overall release of DTX after 28 days was lower compared to DOXO (80 ± 4% vs 85.2 ± 3%), that can be attributed to higher EE (24 ± 2% for DTX vs 17 ± 2% for DOXO).

### E. Computational model

While the experimental determination of drug release in the nanoparticle CQs_7LDD effectively examines the cumulative quantity of DOXO and DTX released over time, a computational model has been developed to aid the understanding of the impact of different design conditions on the drug release behavior of the nanoparticles. This model focuses on drug kinetics and release profiles. Previous research on computational investigation of nanoparticle and nanoparticle kinetics has demonstrated the efficacy of the methods proposed in the referenced physical research.^60^

**FIG. 6.**
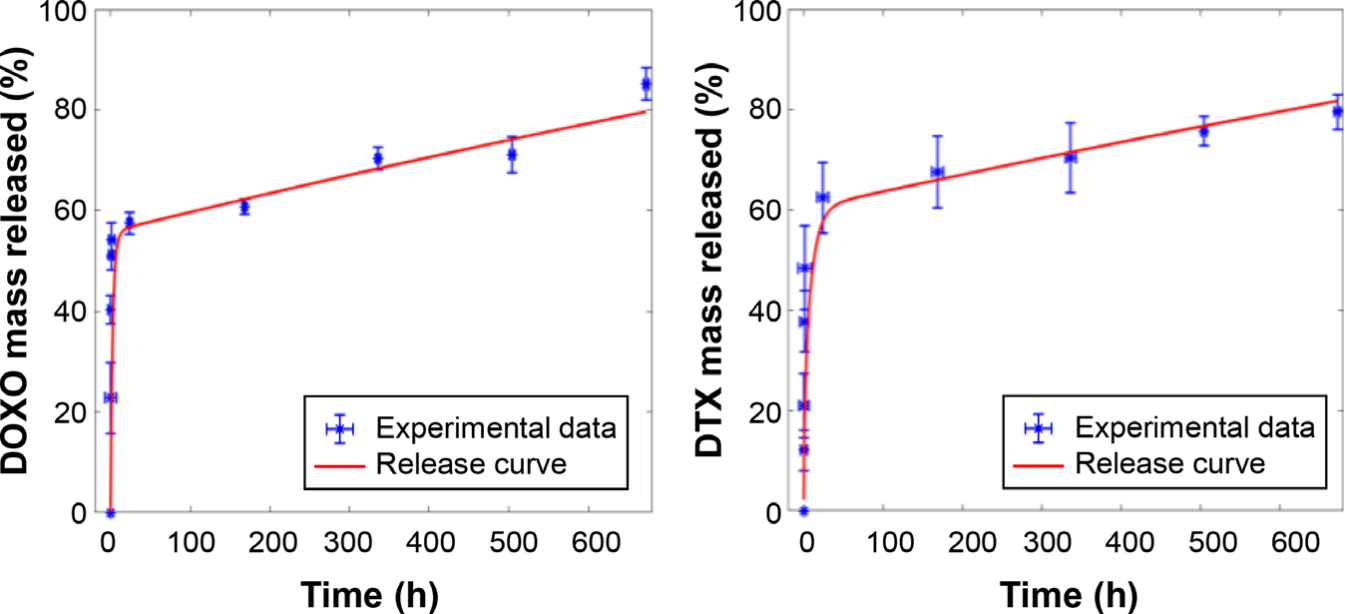
Comparison between experimental data of DOXO (left) and DTX (right) released from CQDs_7LDD nanoparticles (blue points) and computed curves (red line).

In order to assess the reliability and applicability of the proposed model for the experimentally obtained drug release profile, a sensitivity analysis was conducted. This analysis involved varying the conditions to evaluate the impact of different model parameters on the determination of the overall conditions that best match the observed drug release pattern. These specific conditions, which align with the drug release profile, will be further evaluated in the context of nanoparticle drug release.^61^ The comprehensive analysis of the factors derived from the overall model in COMSOL is presented in Fig. S2, which also illustrates the finite element method (FEM) triangular mesh of the computational model. Subsequently, the experimental setup of the nanoparticle was computationally evaluated. The optimal values for the various parameters, obtained to match the datasets for both release curves of DOXO and DTX, are outlined in Fig. 6. Obtaining computational plots based on these data enhances the characterization of the nanoparticle for future use and modification, aiming to optimize its performance. These plots can predict how even slight changes to the shell or core would impact the overall drug release behavior. Consequently, they aid in determining the optimal conditions for the nanoparticle's performance. Their advantage lies in its predictive capability, allowing it to be calibrated and used for drug release prediction in various single and multilayer particles by determining the relevant parameters. This approach reduces the number of experiments and associated costs. However, one of the main challenges lies in estimating the parameter set that ensures drug release tailored to the intended application. Furthermore, the numerical results offer new insights into drug mass transfer and the influence of different parameters, such as particle shape and multilayer configuration, on the drug release mechanism in any release medium.^11^ Therefore, this model can be utilized to identify and optimize processing parameters that guarantee controlled drug release from the nanoparticle over time. The proposed model represent a step-forward in computational approaches for investigating nanoparticle-based drug delivery systems, because the existing literature's present status retains a somewhat qualitative nature where queries pertaining to drug loading, complex stability, and nanoparticle interactions within the surrounding environment have yet to be explored.^62^

### F. Cell tests

**FIG. 7.**
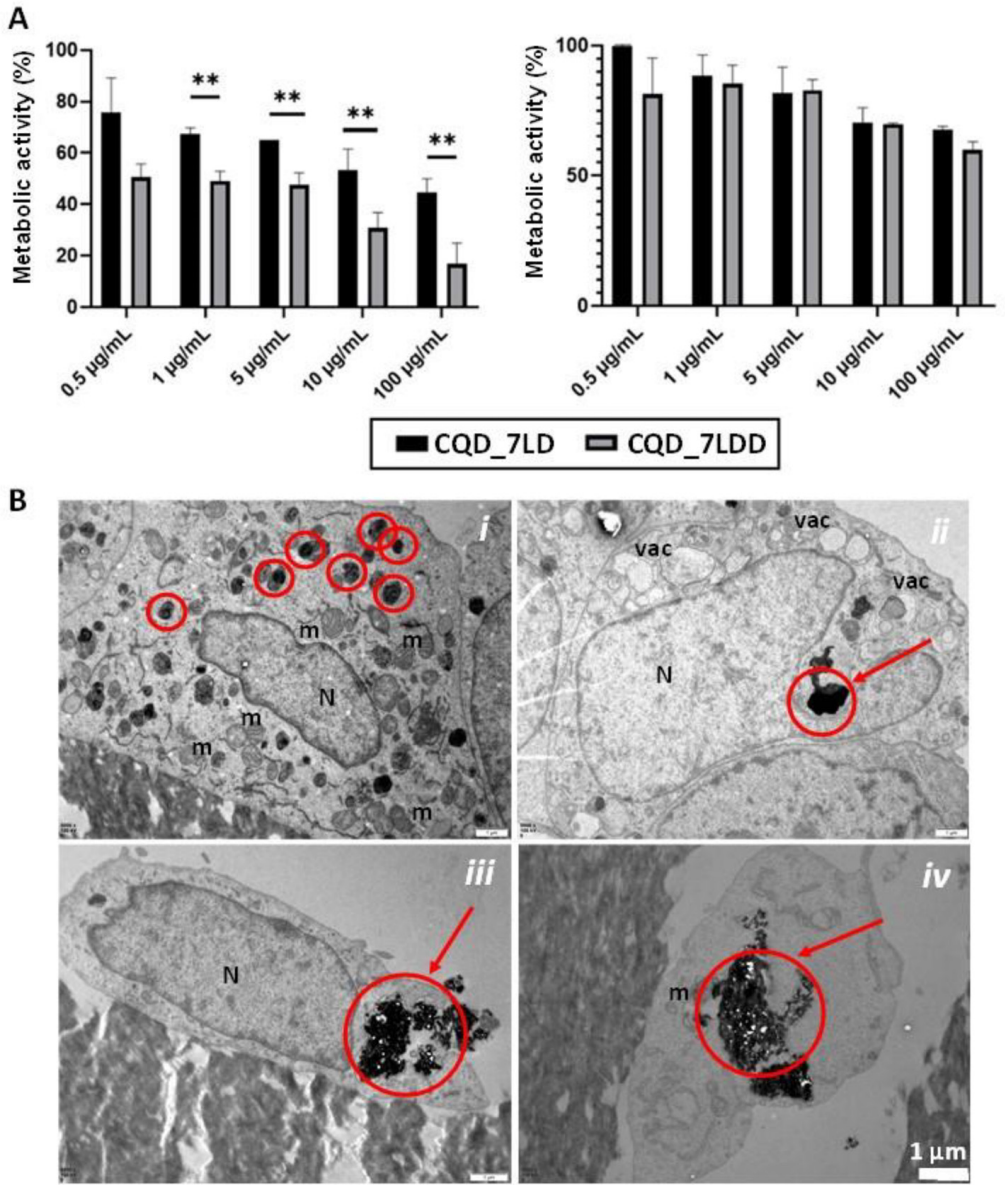
(A) PrestoBlue assay results after seeding Saos-2 cells (left) and U2OS cells (right) with different concentrations of CQD_7LD and CQD-7LDD nanoparticles and (B) TEM images of Saos-2 (i) and (ii) and U2OS (iii) and (iv) cells treated with a concentration of 10 *μ*g/ml CQD_7LDD.

PrestoBlue assay was carried out on the two cell types Saos-2 and U2OS cells after seeding different concentrations of the manufactured CQD_7LD and CQD_7LDD (Fig. 7) for 24 h to test their cytotoxicity. The system CQD_7L was used as control, and the results are available in the supplementary documentation (Fig. S4). Observing both cell types revealed that higher concentrations of the nanoparticles led to lower cell viability, indicating increased cell death. However, it is important to consider the impact of the cell density on cell behavior, as it affects the actual dose of particles reaching each cell. Saos-2 cells exhibited higher cytotoxicity/cell death with CQD_7LDD compared to CQD_7LD. As reported,^63^ current drug delivery preparations of DOXO face challenges in effectively targeting tumors with multidrug resistance. Systems with multiple drugs have shown greater metabolic activity efficiency in delivering drugs to cancer cells compared to single or free drugs.

Previous work incorporating DOXO and DTX into the nanoparticles demonstrated that co-delivery of both drugs-controlled tumor growth better than administering a single drug molecule. This supports the cell viability results and emphasizes the need for both drugs. Particularly, the concentrations of 1 and 5 *μ*g/ml of CQD_7LDD did not show statistically significant differences compared to the 0.5 *μ*g/ml concentration. However, a concentration of 10 *μ*g/ml resulted in a decrease in cellular metabolic activity of 30.7 ± 6.0%, while a concentration of 100 *μ*g/ml showed a decrease in 16.8 ± 8.1%. The latter two concentrations did not exhibit a statistically significant difference. Nonetheless, a concentration of 100 *μ*g/ml is considered extreme for future clinical practice, as concentrations exceeding this have shown little difference and can lead to localization of non-targeting sites.

Statistical analysis revealed a significant difference in efficacy between CQD_7LD and CQD_7LDD for all tested concentrations, indicating the extreme efficacy of the combination of both drugs compared to DOXO alone. Specifically, there was a statistically significant difference between CQD_7LDD and CQD_7LD at concentrations of 10 and 100 *μ*g/ml, with p-values of 0.0325 and 0.0016, respectively. There was no statistically significant difference between the concentrations of 10 and 100 *μ*g/ml of CQD_7LD.

For the nanoparticles seeded with U2OS cells, the results varied significantly, showing a higher cell viability/survival rate compared to Saos-2 cells at identical nanoparticle concentrations. Tests with U2OS cells did not exhibit a significant decrease in metabolic activity when treated with nanoparticles containing both drugs compared to those containing only DOXO. Increasing the concentration from 5 to 10 *μ*g/ml of both systems did not show a statistically significant difference. At a concentration of 100 *μ*g/ml, CQD_7LD and CQD_7LDD resulted in cellular metabolic activities of 81 ± 13% and 86 ± 12% for Saos-2 and U2OS cells, respectively. However, the differences were not statistically significant. The interactions of both systems, CQD_7LD and CQD_7LDD, with Saos-2 and U2OS cells revealed lower cell viability in Saos-2 cells compared to U2OS cells, indicating the higher effectiveness of the nanoparticles on Saos-2 cells. U2OS cells, which do not differentiate or form a calcified matrix, exhibited higher cell viability and survival rates. This aligns with previous studies that show Saos-2 cells' suitability for this type of research due to their high mineralization and proliferation capacity.^64^ For the negative control group, although higher nanoparticle concentrations led to lower cell viability, the results for drug-free nanoparticles (CQD_7L) at different concentrations did not show significant differences (Fig. S4). This is attributed to the absence of drugs in the nanoparticles. For the positive control groups, PrestoBlue assay results for the three different conditions with free drugs (DOXO, DTX, and DOXO/DTX) in Saos-2 and U2OS cells demonstrated almost complete cell death at every concentration, confirming the effectiveness of these drugs against osteosarcoma.

Furthermore, the encapsulation of CQD_7LDD nanoparticles by both Saos-2 cells and U2OS treated with a concentration of 10 *μ*g/ml was analyzed by TEM [Fig. 7(b)]. Transmission electron microscopy (TEM) analysis of both dissected cell lines exposed to nanoparticles for a 24-h duration revealed intracellular particle internalization. A recurrent characteristic of particle systems is their initial uptake by cells through one or multiple endocytic mechanisms after interacting with the cell surface via specific ligand–receptor interactions or nonspecific interactions such as electrostatic and hydrophobic interactions.^65^ In the specific context of CQD_7LDD, electrostatic interactions between the cell membrane and the outer chitosan-based cationic layer of particles facilitate nanoparticles internalization, where the endocytosis mechanism was evident as particles cluster within the cytoplasm, localizing in lysosomes without reaching the nucleus [Fig. 7(b)]. This mechanistic observation is discernible due to significant alterations in cell topography resulting from CQD_7LDD interaction with cell membranes. Notably, observable changes include modifications in cell membrane conformation, nuclear conformation post-endocytosis, and organization and quantity of cytoplasmic organelles, indicative of endomembrane system activation.^66^ Additionally, lysosomes exhibit a darkened appearance due to internalization of CQD_7LDD and the presence of CQDs. Moreover, TEM images revealed that the initiation of CQD_7LDD escape from more labile pinocytic vesicles was observable, facilitating drug release within the cytosol [Fig. 7(b)]. This mechanism induces distortion and damage to cell membrane morphology [Figs. 7(b-ii) and 7(b-iii)] and subsequent disintegration of the cell nucleus, leading to apoptosis or necrosis of cancer cells [Figs. 7(b-ii) and 7(b-iv)]. Indeed, TEM images depicted certain cells undergoing necrosis-induced death due to drug release into the cytosol, resulting in organelle swelling (e.g., endoplasmic reticulum and mitochondria [Fig. 7(b-i)], presence of large vacuoles, plasma membrane rupture [Fig. 7(b-iii)], and eventual cell lysis [Figs. 7(b-ii) and 7(b-iv)].

**FIG. 8.**
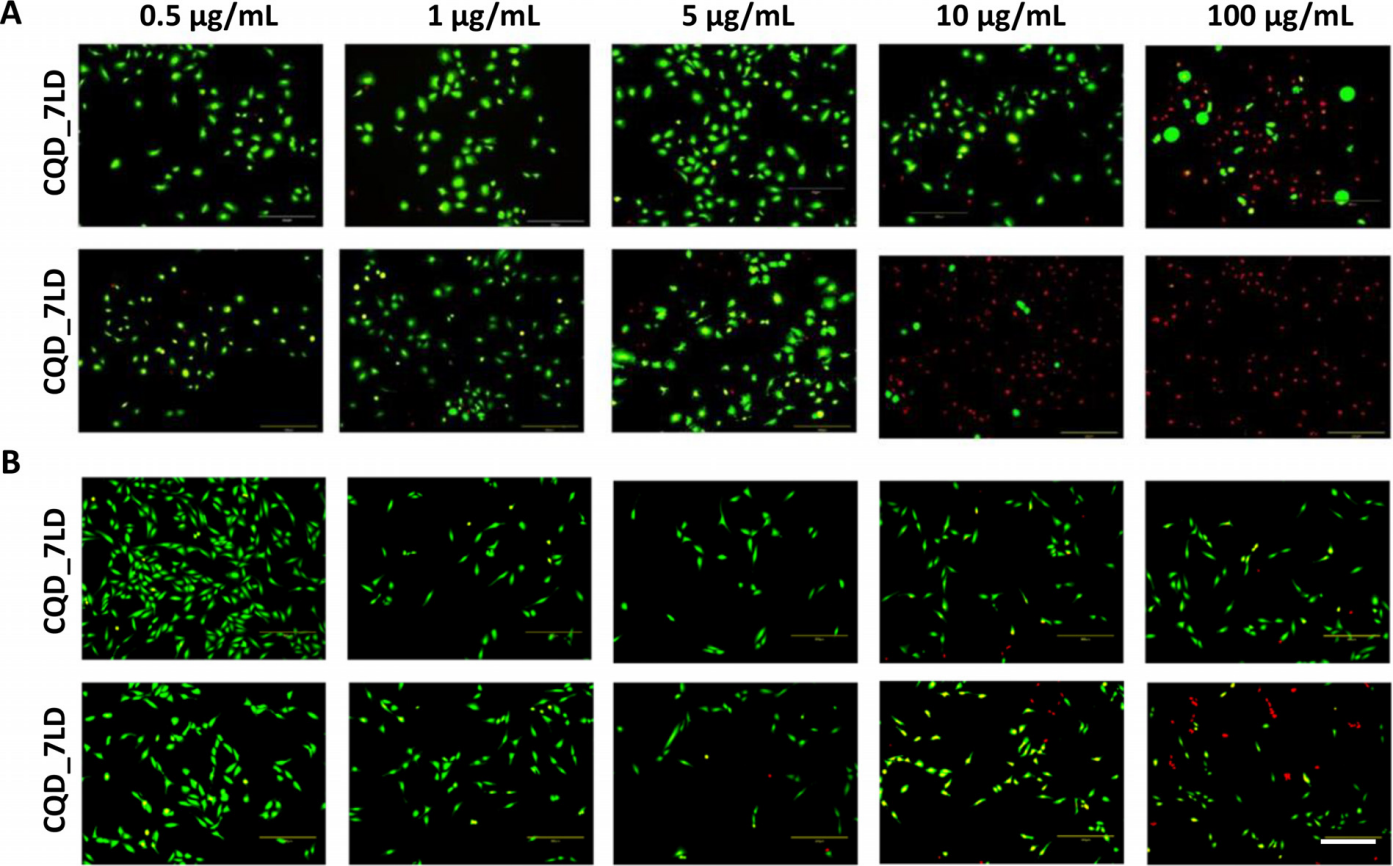
Live/dead images of Saos-2 (A) and U202 (B) cells after 24 h of seeding in the presence of different concentrations (from 100 to 0.5 *μ*g/ml) of the manufactured nanoparticles loaded with DOXO and DOXO/DTX. Scale bar = 300 *μ*m.

To confirm the metabolic activity results, live/dead images (Fig. 8) also supported the observation that U2OS cells were less susceptible to the nanoparticles compared to Saos-2 cells. Additionally, the images of U2OS cells treated with CQD_7LD and CQD_7LDD showed similar viability results, indicating that the presence of both drugs did not significantly affect the survival of U2OS cells.

Finally, active targeting for nanoparticles in the treatment of osteosarcoma is crucial for enhancing therapeutic efficacy while minimizing off-target effects. The modification of nanocarriers with targeting ligands enables precise spatial control *in vivo*, significantly improving the effectiveness of chemotherapeutic agents compromised by passive accumulation and the inability to specifically identify tumor cells.^67^ Various biomarkers are specifically or highly expressed on the surface of tumor cells, and the ligand-modified nanocarrier system can efficiently identify tumor cells by binding to these markers, thereby minimizing damage to normal tissues. Due to its high versatility, LbL assembly allows for the incorporation of various active compounds, including, for example, aspartic acid (Asp)-rich and YSAYPDSVPMMS (YSA) peptides, and the Trastuzumab monoclonal antibody.^68^ These compounds actively target the nanoparticles to osteosarcoma cells, enhancing drug accumulation at the tumor site and amplifying the therapeutic impact of anticancer agents. Thus, the ability to actively target osteosarcoma with nanoparticles holds immense promise for advancing cancer therapeutics, offering a tailored and efficient strategy for combating this aggressive form of bone cancer.^69^

## III. CONCLUSIONS

In this work, in-house synthesized CQDs were organized into a spherical core with DOXO, stabilized with a chitosan nanolayer. The obtained CQDs-loaded cores were then functionalized by LbL assembly for creating a multilayered coating to incorporate a second drug, DTX, in addition to the DOXO. This assembly approach ensured robust stability in physiological conditions, improved synthesis efficiency, potent drug delivery capabilities, and sustained drug cellular. This was validated through comprehensive investigations conducted *in vitro* using two osteosarcoma cell lines, exhibiting remarkable tumor inhibition efficiencies of approximately 70% against Saos-2 cells. Significantly, the *in vitro* outcomes validate the potential utility of the synthesized nanocarrier for medical applications, indicating promise for scalability following rigorous assessments across additional animal models. This holds substantial implications for the development of new drugs and the generation of novel insights into the biological mechanisms underlying osteosarcoma.

## IV. METHODS

### A. Materials and chemicals

For the LbL assembly, chitosan (CH) as polycation and chondroitin sulfate (CS) as polyanion were purchased by Sigma-Aldrich (UK). Doxorubicin hydrochloride (DOX; 98.0–102.0% HPLC, Apollo Scientific Ltd) and docetaxel (DTX, purity > 99.0%, Apollo Scientific Ltd) were the selected drugs incorporated in the LbL-functionalized nanoparticles. Both drugs were dissolved separately in dimethyl sulfoxide (DMSO) (≥99.7%) and phosphate buffer saline solution (PBS) (Sigma-Aldrich, UK) in a volume ratio 1:1, to achieve 10 mM and 600 *μ*M, respectively, and then stored in a freezer set at −20 °C before further use. For the synthesis of the CQDs, chitin (Sigma-Aldrich, UK) was selected as starting biomass. Sodium chloride (NaCl) salt (Sigma-Aldrich, UK) was used and added to the ice bath in the pyrolysis set up to get the carbon dots. Deionized water was obtained throughout Milli-Q^®^ Water Purification System (IQ 7005, Merk, UK).

### B. Chitin biomass processing and synthesis of carbon dots

**SCHEME 1.**
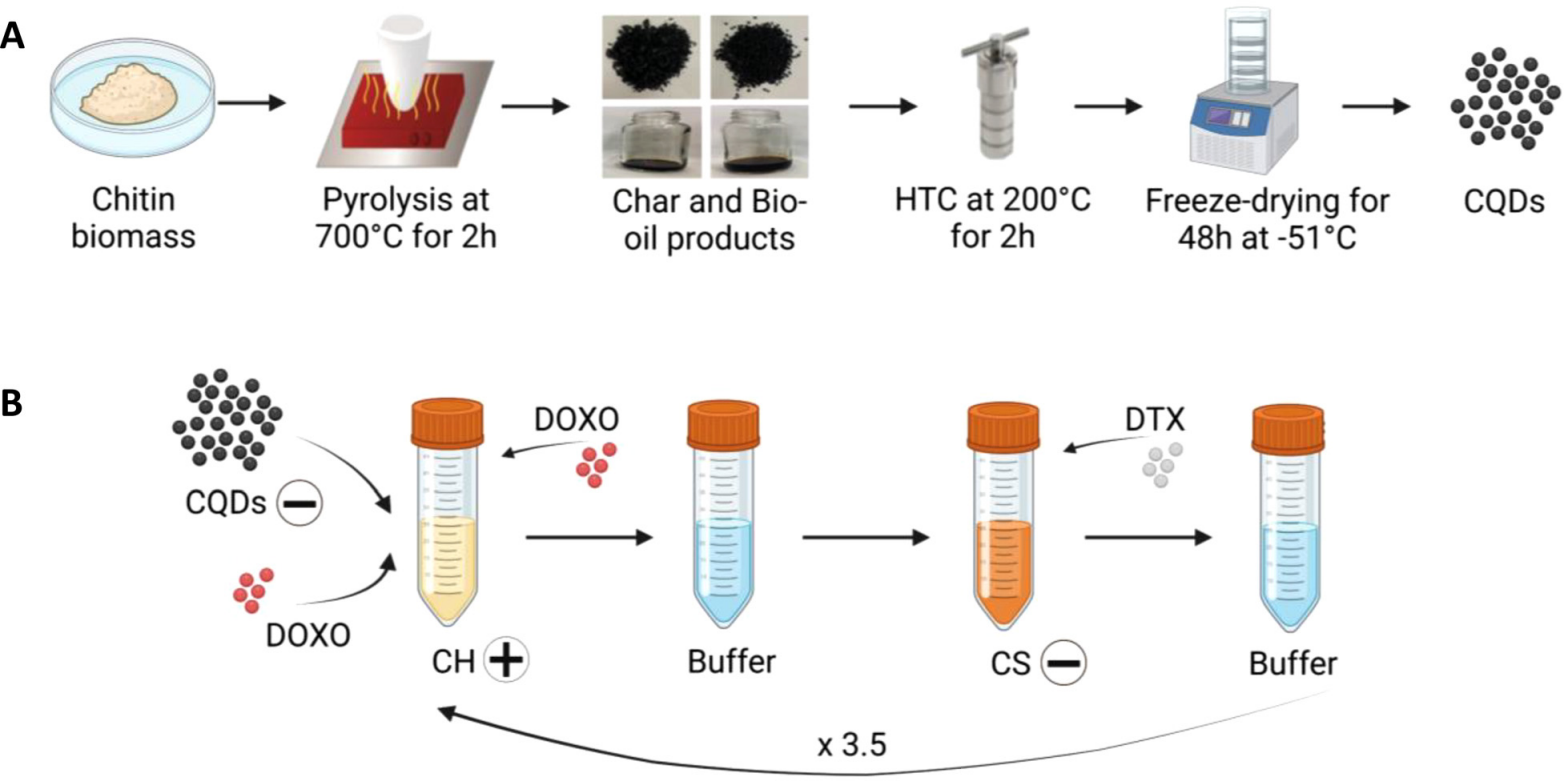
(a) Synthesis procedure of the carbon quantum dots (CQDs) following a bottom–up method consisted in two-step process called pyrolysis-carbonization method to convert the chitin biomass to char (via pyrolysis) and to CQDs (via hydrothermal carbonization, HTC) and (b) LbL scheme for the manufacturing of the functionalizing nanoparticles coated with a multilayered nanocoating consisting of seven layers, where the CH has been used as polycation (positive charged) while the CS as polyanion (negative charged). The CQDs are embedded in the core of the LbL-nanoparticles, while DOXO is incorporated within the CH layer and DTX within the CS layer.

CQDs were prepared by bottom-up method consisting of two-step process called “pyrolysis-carbonization method,” to convert the chitin biomass into carbon-rich materials (known as char) (via pyrolysis) and then to CQDs (via hydrothermal carbonization, HTC) as shown in Scheme 1(a).

The first step involved a pyrolysis treatment using chitin biomass as carbon sources where the chitin was treated in a tube furnace at 700 °C for 2 h in a N_2_ atmosphere. During the pyrolysis reaction, char and bio-oil products are formed. Then, the char products were transferred to a Teflon‐lined stainless-steel autoclave and incubated at 200 °C for 2 h in de-ionized water to obtain CQDs solution, followed by a purification step using 0.22 *μ*m filter papers (Millipore, UK) to remove excess solid and sequential centrifuging at 400 rpm for 20 min (Centrifuge 5701, Eppendorf, UK) was performed. Finally, the obtained CQDs were frozen at −20 °C and then subjected to a freeze-drying process (LD-1 Christ freeze-dryer. UK) for 48 h at −51 °C. CQDs were stored at 4° C protected from light before further use.

### C. Manufacturing LbL-functionalized nanoparticles

LbL assembly protocol [Scheme 1(b)] was conducted using 1 mg/ml of the polyelectrolytes (CH and CS) dissolved previously in sodium acetate buffer (0.1 M, pH 5). The washing steps were carried out in sodium acetate buffer (0.1 M, pH 5). Briefly, a concentration of 0.2 mg/ml negatively charged CQDs dissolved in 1 ml of sodium acetate buffer was used for the formation of the nanoparticle core. Following a centrifugation of the CQDs solution at 10 000 rpm for 10 min (Centrifuge 5701, Eppendorf, UK), the supernatant was replaced by 1 ml of CH solution, acting as polycation, to stabilize the CQD core by deposition of a polymeric nanocoating. The forming solution was then shaken at 80 rpm for 15 min using an orbital shaker (SSM1, Stuart), followed by a first centrifuge of 10 min at 10 000 rpm to separate the formed nanoparticles from the polyelectrolyte and then followed by two washing steps, replacing the supernatant with 1 ml of sodium acetate buffer. For the first washing step, the nanoparticles were shaken again for 10 min at 80 rpm, followed by a centrifugation for 5 min at 10 000 rpm. For the second washing step, a similar procedure was repeated by shaking the nanoparticles for 3 min at 80 rpm and then centrifuged at 10 000 rpm for 10 min. This procedure was repeated for the deposition of next CS polyanionic nanolayer. Before the last centrifugation step, 100 *μ*l of functionalized nanoparticles were collected to measure the corresponding ζ-potential by dynamic light scattering (DLS) (see Sec. IV D). For the incorporation of the drugs, an aliquot of 34 *μ*l of DOXO solution (10 mM) was added in 10 ml of cationic CH polyelectrolyte, while 34 *μ*l of DTX solution (100 *μ*m) was added to anionic CS polyelectrolyte in order to incorporate the drugs within the positive and negative layers, respectively. The last layer did not contain any drug. The LbL procedure was repeated until the formation of a multilayered coating consisting of seven layers. The number of layers was optimized by following a design of experiments to achieve the optimal concentration of drugs encapsulated into the nanolayers after setup and validation of a computational model (see Sec. II E). As a final step, the coated nanoparticles were freeze-dried (LD-1 Christ freeze-dryer, UK) and stored in a vacuum desiccator for future analysis.

### D. Physico-chemical characterization of the LbL-functionalized nanoparticles

#### 1. Fourier transformed infrared spectroscopy (FTIR-ATR) analysis

The infrared spectra were obtained with a Spectrum Two PE instrument equipped with a horizontal attenuated total reflectance (ATR) crystal (ZnSe) (PerkinElmer Inc., US). The analyzed samples were placed directly on the ATR crystal, and the spectra were collected in absorbance mode and recorded in the wavelength range of 4000–550 cm^−1^. Each spectrum was the result of averaging 32 scans with a resolution of 4 cm^−1^.

#### 2. Surface charge measurement

A Zetasizer Nano ZS Instrument (Malvern Panalytical Ltd) was used to measure the surface charge of the nanoparticles during the formation of each layer. The samples (aliquots of 100 *μ*l collected after the first washing step for each layer) were diluted 1:10 in dH_2_O. The values were as result of the average of three measurements where each measurement was obtained after a maximum of 100 runs. The experiments were performed in triplicate.

#### 3. Efficiency of encapsulation of DOXO and DTX in the nanoparticles

For all the nanoparticles containing drugs, the encapsulation efficiency (EE) was determined. Upon the addition of every layer, the supernatant of the successful layer addition was recovered and 100 *μ*l of this solution was transferred into a 96-multiwell in triplicate. Following this, each of the triplicate of the samples was analyzed in absorbance using a FLUOstar Omega MicroPlate Reader (BMG Labtech, UK), measuring separately the absorbance of the unencapsulated DOXO (at 480 nm) and DTX (at 230 nm), subtracting the average absorbance of the three wells containing the blank (washing solutions or the drug-free deposition solutions). Then, from the measured absorbance, the corresponding DOXO and DTX concentration in *μ*g/ml were calculated by using corresponding drug calibration curves, previously created (Fig. S1). The determination of the total mass of drug encapsulated within the layers (EE) is determined as follows:

$$EE(\%)=\left(\left(\text{amount}\;\mathrm{of}\;\text{drug}\;\text{added})-(\text{amount}\;\mathrm{of}\;\text{drug}\;\text{not}\;\mathrm{in}\;\text{nanoparticles}\right)\right)/\left(\text{amount}\;\mathrm{of}\;\text{drug}\;\text{added}\right).$$ (1)

#### 4. Process yield (Y)

For each of the nanoparticles derived, the process yield was the measurement of formulation produced (%) after freeze drying of the final nanoparticles, as follows:

Y(%)=Weight of produced nanoparticles/(Sum of the weights of all starting reagents). (2)

Produced formulations were measured following freeze-drying of solutions containing the nanoparticles coated with the multilayer coating incorporating the drugs.

#### 5. Transmission electron microscopy (TEM) analysis

TEM was employed to determine the morphology and size of the nanoparticles, after addition of each layer to track their dimension change. The analysis was performed employing a Philips CM 100 Compustage (FEI) transmission electron microscope (Philips) at HV = 100.0 kV, and the digital images were captured using an AMT CCD camera (Deben) with a range of magnification up to 130 000×.

#### 6. Drug release studies

For the determination of drug release profiles, 0.4 mg of nanoparticles were accurately weighted, dispersed in 1 ml PBS (Sigma-Aldrich UK) and incubated at 37 °C for up to 28 days. PBS was used as release medium. The amount of drug released was measured at specific time points. In order to characterize the initial burst effect, the measurement was taken after 10 min and up to 6 h soaking during the first day of incubation. At each step, 60 *μ*l of supernatant (replaced at each withdrawal with an equal amount of PBS) was taken from each sample and mixed with PBS in a 1:10 ratio to get a final volume of 600 *μ*l. Next, the obtained solution was centrifuged at 13 000 rpm for 10 min and the supernatant was taken and transferred to a new Eppendorf tube. Finally, the optical density of the solution was measured separately at 480 nm for DOXO and at 230 nm for DTX using a FLU Ostar Omega MicroPlate Reader (BMG Labtech). For each time step analyzed, the effective absorbance was calculated by subtracting the average absorbance of the wells containing the blank (PBS). Through the two different calibration curves of DOXO and DTX in PBS, the concentration of DOXO and DTX released at each time step (*μ*g/ml) were obtained; multiplying the concentration by the volume of the supernatant (1 ml), the mass of DOXO and DTX released were obtained. A cumulative release plot was calculated, and the experiment was done in triplicate.

#### 7. Fluorescence analysis

To evaluate the fluorescence behavior of the manufactured nanoparticles, the absorption spectra were recorded on a Jenway 7315 Spectrophotometer, while the fluorescence measurements were performed on Shimadzu RF-6000 Spectro fluorophotometer. The spectra were measured with a resolution of 1 nm^−1^.

### E. Computational model on drug kinetics release from multilayer nanoparticles

The data obtained from the *in vitro* drug release experiments (DOXO and DTX) were used to setup and validate a mathematical model implemented for drug release from nanoparticles. The mathematical model was implemented from previous work by Barchiesi *et al.*^11^ This mathematical model, originally computed using the commercial software COMSOL Multiphysics, schematically represents the nanoparticles as having an inner core and a single polymeric shell representing, as a whole, the multilayered nanocoating [Fig. S2(a)]. The internal core of CQDs is denoted as Ω_0_ while the single, equivalent, layer modeling the seven outer layers of the considered nanoparticles is denoted as Ω_1_. While the combined thickness of these layers is much smaller than the core radius, they still present significant resistance to drug flux due to the numerous chemical bonds and encapsulation. Previous research has demonstrated that these layers act as a shield, preventing a complete burst release.^12^ According to previous research,^15^ the modeling of drug dissolution in the core requires the introduction of nonlinearities. In the adopted modeling, each drug has been considered encapsulated within the core dissolves at a specific rate *β*, which is proportional to the difference between the concentration of the dissolved drug and its solubility *S* in the physiological solution. Once dissolved, the drug can diffuse through the core with a diffusion coefficient *D*_0_. The dynamics of drug dissolution and diffusion in *Ω*_0_ are defined by following nonlinear partial differential equations:

$$\frac{\partial b_{0}}{\partial t}=-\beta b_{0}^{\alpha }(S-c_{0})\;\text{in}\;\Omega_{0},$$ (3)

$$\frac{\partial c_{0}}{\partial t}=\nabla (D_{0}\nabla c_{o})+\beta b_{0}^{\alpha }(S-c_{0})\;\text{in}\;\Omega_{0},$$ (4)

which fulfills mass conservation and where the unknown field *b*_0_(*x*, *t*) is the concentration of the undissolved drugs within the core and the field *c*_0_(*x*, *t*) is the concentration of the dissolved drug. The parameter *β* is the specific dissolution rate and *S* is the solubility of drugs in PBS. Furthermore, in these equations, the symbol *∇* represents the gradient operator, and the exponent α considers possible effects on the dissolution rate due to variations in the particle surface area. In literature, it has been reported that for spherical nanoparticles of this nature, *α* is equal to 2/3.^13^

Denoting the bound and unbound phase concentration fields in the shell *Ω*_1_ with *b*_1_(*x*, *t*) and *c*_1_(*x*, *t*) [Fig. S2(a)], the dynamics of the drugs in *Ω*_1_ can be represented by the equations below, where no interactions between the drugs are considered and mass conservation is still fulfilled,

$$\frac{\partial c_{1}}{\partial t}=\nabla (D_{1}\nabla c_{1})-kc_{1}\;\mathrm{in}\;\Omega_{1},$$ (5)

$$\frac{\partial b_{1}}{\partial t}=kc_{1}\;in\;\Omega_{1},$$ (6)

where *D*_1_ is the diffusion coefficient in the coating shell. Experimental evidence shows that a fraction of the initial loaded drug is retained in the shell and is never released.^18^ We model this aspect in the above equations by using first-order reaction kinetics, where diffusing the drug through the shell can potentially be permanently bound at a rate *k*. Contrarily to what has been observed and modeled in the previous work,^11^ before reaching a value that remains constant in time definitely, the released mass percentage increases almost linearly after the initial burst release. This is mainly due to dissolution/degradation of the coating and the release of the embedded payload into the surrounding environment. To model such a linear phase, a moving boundary problem has hence been used, namely, each point of the external boundary of the domain *Ω*_1_ has been considered to move toward the core with (known) constant radial velocity. Such a velocity has been used as the same for each point, so that the resulting transformation of the boundary is a contraction. To close the system Eqs. (3)–(6) proper interlayer and boundary conditions are imposed.^18^ As initial conditions, the drugs are homogeneously distributed initially, and their release will be hindered by the resistance offered by the layers. At the outer surface, it was imposed a perfect sink condition, to mimic the *in vitro* experiments conditions where the nanoparticles are immersed in a large environment fluid. The setup of the initial parameters for the developed model in COMSOL are reported in Fig. S2(b).

### F. Cell biological evaluation of the LbL-functionalized nanoparticles

#### 1. Cell culture and seeding

Saos-2 and U2OS osteosarcoma cancer cells were purchased from Sigma-Aldrich (UK) and cultured as recommended by the seller. Briefly, both cells were grown at 37 °C, 5% CO_2_, in Dulbecco's Modified Eagle Medium (DMEM, Sigma) supplemented with 10% fetal bovine serum (FBS) and a 1% antibiotic mixture containing penicillin and streptomycin (100 U ml^−1^).

#### 2. Cell viability and metabolic activity

Solutions containing the developed LbL-functionalized nanoparticles at different concentrations (from 0.5 to 100 *μ*g/ml) were prepared by dissolving the nanoparticles in DMEM and then sterilized by filtration through a 0.22 mm Millex GP PES membrane syringe-driven filter unit (Millipore, SLS, UK) using 5 ml plastic syringes. U2OS and SAOS-2 osteosarcoma cancer cells were seeded in a 96-well plate (7000 cells/well) and allowed to grow for 24 h. The following day the media was removed and replaced with solutions containing the drug-loaded nanoparticles or free drugs as positive control.

After 48 h of incubation, cell viability was assessed with the live/dead staining (LIVE/DEAD^®^ Cell Imaging Kit, Life Technologies, Thermo Scientific, US). According to the manufacturer's protocol, each well was washed with PBS and stained with 150 *μ*l solution of 4 *μ*M Ethidium homodimer-1 and 2 *μ*M calcein in PBS. After 30 min of incubation at room temperature, cells were imaged with a EVOS M5000 fluorescence microscope to detect calcein (ex/em 488 nm/515 nm) and Ethidium homodimer-1 (ex/em 570 nm/602 nm), respectively. Furthermore, at the same time point, Presto Blue assay was exploited to test the metabolic activity of cells seeded with the different diluted nanoparticles or free drug solution. A Filter-based FLUOstar^®^ Omega multimode reader (FLUOstar^®^ Omega, Germany) was used to measure the fluorescence (560 nm excitation and 590 nm emission) after 1.30 h of incubation with a 10% aliquot of Presto Blue (Thermo Scientific, USA). The results were expressed as mean ± standard deviation.

Finally, uptake of the nanoparticles by SAOS-2 cells was verified by TEM analysis. Particularly, 5000 cells were kept adhering for 24 h on a 24-well plate having Corning™ Transwell™ Multiple Well Plate with Permeable Polyester Membrane Inserts (Thermo Scientific™). Then, the nanoparticles were incubated for 24 h. Following removal of the culture medium and washing in PBS (three times), the cells were fixed on the membranes using a pre-warmed solution of 2% glutaraldehyde (TAAB Laboratory Equipment) in sodium cacodylate buffer at 4 °C. After various dehydration steps, the cell layer was embedded in resin, and cut in ultrathin sections using a diamond knife on a Leica EM UC7 ultra microtome (Leica Microsystems). The sections were stretched with chloroform to eliminate compression, mounted on Pioloform-filmed copper grids (Agar Scientific) and ready to be visualized using the TEM equipment described above in Sec. IV D.

### G. Statistical analysis

Tests were performed at least in triplicate for each sample. The results are presented as means ± standard deviations. Statistical significance was evaluated by analysis of variance (ANOVA), using GraphPad Prism software, followed by Turkey's multiple comparison test using levels of statistical significance of p < 0.05 (^*^), p < 0.01 (^**^), p < 0.001 (^***^), and p < 0.0001 (^****^).

## SUPPLEMENTARY MATERIALS

See the supplementary material for the calibration curves of docetaxel and doxorubicin drugs (Fig. S1), the input parameters for the setup of the COMSOL model (Fig. S2) alongside the FTIR-ATR chemical characterization of the CQD_7L nanoparticles (Fig. S3), and their influence on the metabolic activities of Saos-2 and U2OS cells.

## Data Availability

The data that support the findings of this study are available from the corresponding authors upon reasonable request.
